# Remarks on the Inability to Produce the Effect of Emetics in Horses

**Published:** 1810-10

**Authors:** George Hargrove

**Affiliations:** Member of the Roy. Col. of Surgeons, London; and Assist. Surg. Royal Reg. of Artillery


### *Remarks on the inability to produce the effect of Emetics in Horses.* By GEORGE HARGROVE, Member of the Roy. Col. of Surgeons, London; and Assist. Surg. Royal Reg. of Artillery.

COMPARATIVE anatomy possesses so many sources, not only of information to those inclined for its reception, but of philosophical amusement to the otherwise indifferent student, that one feels surprized it is not more the order of the day, or at least, that some trifling portion of our time is not more generally devoted to an enquiry into that order of animated nature, which in every step so strongly exhibits our own wonderful structure; and at the same moment that it inspires us with the most profound reverence, for the magnanimous author of contrivances so truly noble, it imperceptibly leads leads on the reflecting mind to the knowledge of phenomena, still more interesting to our limited conceptions. Ambition for knowledge has, time immemorial, been the great characteristic of man; and as long as he restrains this inherent principle within reasonable bounds, he only complies with the ordinance of Providence, and by this means, amply enriches his intellectual powers; but the instant he exceeds those limits, he audaciously pries into secrets, which his comprehension does not authorize him to understand, and, from his too ardent, I might add, criminal presumption, he often sacrifices the reasoning faculties he previously possessed, to an inconsiderate zeal after works, that are only familiar to the most exalted genius.

Having often heard it argued by literary men, both before and after I entered on the the studies of my profession, that it was impracticable to produce the effect of emetics in horses, even if this description of medicine should be given in the largest quantities; I determined to let no opportunity pass unnoticed, that would be likely to develope the mystery, and although the majority of my *professional* readers may not feel interested in this communication, yet, as there is a chance that some few may be unacquainted with it, especially some of *those* who are in the habit of perusing this valuable publication, *without belonging to the profession*, I feel an equal interest in offering it, and can only say, that in addition to the latter circumstance, a zealous desire of diffusing, even so inconsiderable a portion of physiological observation as this is, are to me sufficient inducements for wishing to see it in print. I lately had two very favorable opportunities of examining the situation, form, and structure of the stomach of a horse; one of these animals had been shot in consequence of being affected with glanders, the other with a disease, which this class of beasts appear extremely liable to, lock-jaw. After having the stomach and its appendages, the æsophagus, and part of the duodenum, removed from the abdomen, and cleaned, I carefully made an incision on the greater arch, at the part where the duodenum joins, so that I could with facility introduce my hand without dilating the incision; I now thought to push the index finger of my right hand gently into the cardiac orifice, from the great sac upwards, of course, but to my astonishment, I found it impossible; I refrained from using force, withdrew my hand, and extended the incision to within three inches of the cardia, when I saw the orifice firmly contracted; I now used a little force, and got my finger beyond the stricture, which girt it round with an unusual degree degree of firmness and tightness. After having satisfied myself as to the cause, I divided the stricture, and consequently, the cardiac orifice longitudinally, with one of Mr. Pott's bistouries, which I had nearly broken from the force it required, and the depth of substance to cut through. I now cautiously dissected the villous coat off, and distinctly ascertained a true sphincter muscle, situated transversely with respect to the direction of the œsophagus next the stomach: on exposing this mass of muscular fibres still more, I observed it would not be less than two inches in circumference, and that it appeared to be intersected in various places by irregular portions of ligament, exactly as we perceive in the "straight muscles" of the human abdomen. I could plainly feel, through all the coats and attachments of the stomach, that the cardiac orifice was considerably thicker and harder than any other part in its vicinity. The action of this muscle on my finger can easily be accounted for, when we consider the well known vital property that subsists for some time after death, and which appears to be peculiar to muscles; it is also known to be a power totally independent of the will of the animal, as is evinced in almost every surgical operation.

I remarked, during the dissection of the horse's stomach that had been shot in consequence of *tetanus*, that the sphincter muscle was not only impervious to my little finger, but to water, which it visibly closed on, shortly before being taken from the abdomen; which is a convincing proof that even after the wretched animal had been deprived of life, this identical muscle, which participated so remarkably in depriving the stomach of its natural stimulus, retained for a period of thirty minutes the morbid function it had acquired on the accession of the disease. This stomach was nearly empty, the miserable creature not having been able either to eat or drink for above forty-eight hours prior to his death.

I should have mentioned, that I never observed the rigidity of muscular fibre, any thing approaching to the extent it did in this horse, in a human being labouring under tetanus.

The ideas I was impressed with when I first observed this rare and curious structure, after a little consideration, yielded to others of a more rational description, for although I had many times observed a similar effect to that of emetics produced in other quadrupeds, for instance, in dogs when sea-sick, yet it did not immediately occur to me, that that Providence who pays attention to the comforts of what we esteem the vilest of beings, even in this instance, evinces the peculiar regard she has for human nature, by depriving one of the noblest animals of that convulsive effort, (so natural in most other classes) to expel the molesting cause of sickness, in order that his labours may be still more subservient to the purposes of man. We can easily imagine what would be the case during a long and dreary journey, when, I have no doubt, those poor creatures, that we so often see abused, suffer many a pang from temporary illness, but which nature takes care they surmount, though in the midst of their toil, by stimulating the other emunctories of the body to a degree of action, equal to the removal of the offending cause.

P. S. Agreeably to the intention I expressed in the case of William Smith, serjeant in our regiment, in the publication of May last, I beg leave to mention, that a very extensive exfoliation has taken place from the parietal bones since, and I understand he continues in tolerable health, and suffers no pain. I had an opportunity, about a month since, of seeing one piece of the detached bone, which appeared to be two inches in circumference.

*Ringmer, near Lewes, Sept.* 5, 1810.

